# Examining the maternal health services-seeking behaviour among adolescent urban refugees in Kampala, Uganda

**DOI:** 10.4314/ahs.v23i1.41

**Published:** 2023-03

**Authors:** Olivia Nakisita, Charity Kirabo-Nagemi

**Affiliations:** 1 Makerere University College of Health Sciences, School of Public Health; 2 Centre for Sexual Reproductive Health and Rights, Makerere University College of Health Sciences, School of Public Health

**Keywords:** Adolescent health, refugees, maternal health services

## Abstract

**Background:**

Reproductive health is a key concern during crisis situations and improving maternal health is crucial. In Uganda, refugees living in urban areas are expected to be self-reliant and this makes the adolescents who get pregnant vulnerable.

**Objective:**

To investigate the maternal health services seeking behaviour of the adolescent refugees in Kampala.

**Methods:**

This study examined the experiences of twenty refugee girls who got pregnant and delivered babies from the ages of 10 to 19, while living in Kampala. This was done through in-depth interviews. Thematic content analysis was used to interpret, code and analyze the data.

**Results:**

This study found that many of the refugee girls get pregnant as a result of sexual violence. The girls seek maternal health services from the public and private facilities around Kampala, but face barriers to access and utilization like poverty, language barrier, health worker's attitudes, and overcrowded facilities and therefore many received suboptimal care.

**Conclusion:**

The urban refugee adolescents who get pregnant face several challenges to utilize maternal health services. Increased funding, favourable policies and programs are needed to support these girls to access comprehensive maternal health services because they face multiple vulnerabilities.

## Introduction

Worldwide there are 68.5 million forcibly displaced people and 25.4 million of these are refugees^1^,^2^. Uganda hosts approximately 1.4 million refugees and 5.2% (68,318) of the refugees are living in Kampala1. They hail from Sudan, South Sudan, Congo, Burundi, Rwanda, Somalia, Ethiopia, as well as Eritrea and more are settling in every day, according to the UNHCR. Sexual, Reproductive Health and Rights (SRHR) are essential parts of everyone's life, including the lives of refugees^1^.

Adolescents are vulnerable to rape and sexual exploitation due to their lack of power and resources, while some of them resort to selling sex to meet their needs and family needs^1^,^2^. Most of the adolescent girls are at risk or already struggling with the consequences of an unplanned pregnancy^1^,^2^. Adolescents are also at a high-risk during pregnancy and child birth, and this is the second cause of death among 15- to 19-year-old girls globally^1^.

Adolescents as a group are however often ignored during emergencies, looked at as not vulnerable, and their needs consequently being overlooked^2^,^3^. Among the reasons that affect the views, perceptions and behaviour of the young people regarding relationships and marriage are; the disruption of families during emergencies, poverty and the lack of education. Although there are policies, guidelines and standards for SRHR service delivery during emergency situations, SRHR is overlooked, underfunded or deprioritized^1^–^5^. There is need for an evidence base for the SRHR interventions in humanitarian crises^1^.

This study sought to generate evidence on the seeking behaviour for maternal health services such as antenatal care (ANC), delivery, post abortion care (PAC) and post natal care (PNC), by the adolescent refugees who are live in Kampala, Uganda.

## Methods Study Design

This was a cross-sectional study which utilized qualitative methods.

### Study Area

The study was carried out in Wakaliga, Busega, Nateete, Kasubi, Katwe, Salama, Konge, Kikoni and Mpererwe areas within Kampala district.

### Study period

The interviews were held between August 2020 and January 2021.

### Study Population

The study was executed among refugee adolescent mothers from Sudan, South Sudan, Congo, Burundi, and Rwanda, who are registered with the Office of the Prime Minister as urban refugees.

### Inclusion Criteria

The study population were girls who had got pregnant or delivered babies while they were 10-19 years old, from 2017 to 2021 and this was regardless of the outcome. The outcomes were defined as positive where the adolescent mother had a safe delivery and the baby lived and negative outcome where one had suffered a miscarriage or the baby died within a week of delivery.

### Exclusion Criteria

This study excluded the girls that delivered babies while living outside Kampala, as well as in refugee settlements and those that did not consent to take part in the study.

### Sample size determination and procedure

The respondents were selected purposively, their representation is in Table 1 below.

**Table 1:** Respondents categories

Country of origin	Criteria	No. of respondents (n)	Percentage by country of origin
Sudan	Positive outcome (3)	3	15%
South Sudan	Positive outcomes (3)	3	15%
Congo	Positive outcomes (6)	6	30%
Burundi	Negative outcomes (2) Positive outcomes (4)	6	30%
Rwanda	Still pregnant (1) Had a positive outcome (1)	2	10%
Total		20	100%

### Instruments

An interview guide was used to conduct the In-depth Interviews with the adolescent mothers. Twenty In-depth Interviews were conducted by well-trained research assistants until a point of saturation.

### Data Management and Analysis

All interviews were recorded electronically and transcribed verbatim. Codes were prepared manually using themes determined from literature and the objectives of the study. The key themes in the text were analysed in-depth to understand the circumstances under which girls got pregnant and whether or not they sought maternal health services that include ANC, Delivery PAC and PNC. Quotes representing the typical views of the adolescent mothers were used to support the themes identified.

### Ethical Considerations

Ethical approval of the study was sought from Makerere

### University School of Public Health Higher Degrees and Ethical Committee

Permission to conduct the study among the refugee population was sought from the Office of the Prime Minister - Refugee department.

Voluntary participation in the study was sought with a written or verbal informed consent and the participants were informed of their right not to participate if they do not feel like.

Confidentiality was ensured by having face to face private interviews and the respondents remained anonymous their names were not recorded but code numbers were used.

## Results

Social demographic characteristics of respondents A total of twenty (20) refugee adolescent girls were interviewed. This study revealed that some girls got pregnant at as early as 14 years, while others were married by the time, they turned 18. Many of the girls conceived as a result of sexual violence and exploitation and did not have support from a partner during pregnancy. Some of the girls lived without parents or relatives. A good number of the adolescent girls dropped out of school at primary and secondary level and did not have any form of employment (Table 1 below).

**Table 1:** Socio demographic characteristics of the respondents

Variable	Frequency N=20	Percentage (%)
**Age at pregnancy(years)**		
10–14	1	5.0
15 -19	19	95.0
**Marital status**		
Married	4	20.0
Single	16	80.0
**Occupation**		
Employed	3	15.0
Unemployed	17	85.0
**Education level**		
None	0	0.0
Primary	7	35.0
Secondary	13	65.0
Tertiary	0	0.0
**Number of years in Uganda**		
Less than 2 years	0	0.0
2–5 years	12	60.0
6 years and above	8	40.0

### Knowledge of maternal health services

Although most of the girls were aware they had to visit the health facility for antenatal care, deliver at a health facility and attend postnatal care, they were not aware of the post abortion care services at the facilities. The girls who knew where to seek the maternal services reported to have got this information from the people around them and organisations that serve urban refugees.

“*It was Inter-Aid Uganda, they always tell us in the address they give in the morning that if you are sick you go to KCCA Kitebi, Kawaala, Kisenyi and if you have other illnesses Kisenyi can refer you to Mulago or Kiruddu*”

### Antenatal Care seeking behaviour

All the girls interviewed attended antenatal care, however most did not go for all the recommended times and many started ANC late in their pregnancies.

“*I did not know that I had to go for ANC because every time we would go to different hospitals. That's why in the last hospital where I delivered, I was asked for the papers for ANC and I asked them what they mean, and what is antenatal, then they started giving me but I was already 9 months*”

### Delivery services seeking behaviour

This study found that all the refugee adolescent girls that were interviewed had delivered babies at a health facility. Majority of them delivered babies at government facilities that offer free medical care and others at a private facility. The refugee adolescent girls who had their parents and family around had better experiences during delivery compared to the unaccompanied ones.

“*I was rushed to IHK Kabalagala because my condition was not good, the clinic I was visiting for ANC could not handle my condition and I was referred to IHK because I needed a surgery*”

### Postnatal care seeking behaviour

The girls reported that when being discharged from the health facility after delivery, they were asked to return and most of them did. None of the girls mentioned a home visit from a community health worker (VHT). A few of the girls did not return to the health facility because they did have transport.

“*After 2 weeks they told me come to back. We went back and they checked the baby and they said he is ok*.”

Challenges the adolescent girls face while seeking care Transport was a challenge to most of the girls and some walked long distances to the government health facilities, while others could not return for health visits. Most of the girls used a boda-boda (motorcycle taxi) to get to the health facility when in labor and one of them arrived too late when the baby had already died in the womb. Some of the girls went to deliver babies at a private health facility nearby because they could not access the government facility easily at night.

The adolescent girls waited for 3 to 4 hours before they could get services, especially in the public facilities. They said this was because the health workers waited for as many mothers as possible to arrive before they could give a health talk and then offer ANC services. In some facilities the drugs were not adequate and essential equipment such as scans were missing.

“*.... they do not give me all the medicine they give me for few days... I would go and buy in the clinic...... It is expensive because it is 5,000shs and at times you do not have that kind of money*”

The attitude of some health workers discouraged the girls from going back for the services. Some health workers asked for bribes from the girls and other girls reported that they felt worthless by some of comments the health workers made.

“*Yeah, one of them was taking my blood pressure she asked me how old I was I told her 16 years and she started laughing and saying you are supposed to be in school*.”

Language barrier was also reported to be a big challenge faced by the refugee girls who were seeking the services. In most facilities English and Luganda are used to communicate and yet the girls do not understand these languages very well.

“*The health workers were not taking care of every pregnant woman equally. If you speak another language like us Congolese, we speak our languages everywhere you were not served like others. I was supposed to speak Luganda*.”

## Discussion

This study revealed that many refugee adolescent girls living in Kampala face sexual violence such as rape and defilement. This study concurs with a study carried out in Uganda among adolescents which revealed that adolescents have sexuality problems such as unwanted pregnancies, defilement and rape^2^. Another study carried out in the slums of Kampala reveals that there are very high levels of sexual abuse^3^. This is further supported by a study which concluded that the SRHR risk for the urban poor are severe^4^.

Most of the refugee adolescent girls live on their own or with friends and not parents, which puts them at risk. Most of the adolescent girls have no means of livelihood and are more likely to get male partners to support them. When they get pregnant, the partners abandon them. The girls that live with their parents are not better off because their families are poor and have many dependants to care for. Consequently, these girls are forced to look for ways to support their families and end up in early relationships that result in unwanted pregnancies. This finding relates to previous studies which concluded that the disruption of families puts adolescents at a higher risk of early sexual intercourse.^3^,^5^–^7^.

The refugee adolescent girls who get pregnant utilize the antenatal care services, deliver babies at health facilities with skilled care and return to the facilities for post natal care. Most of these girls however reported late for their first ANC visit and did not attend the recommended times. This finding concurs with a study on pregnant refugee women in Syria that concluded that the standards for ANC were not being met^8^.

There is inadequate access to information about the range of services that are available. Most girls were not aware that post abortion care and services for survivors of rape were among the services provided at the health facilities. This finding agrees with studies that concluded that lack of adequate information is a barrier to the utilization of the SRHR services^9^–^11^.

The government facilities that offer free services are few in Kampala, yet the private facilities that are within reach charge exorbitant fees. Consequently, the refugee adolescent girls find it difficult to access the services. This finding concurs with another which concluded that the health services available for the public are limited, as a result small private facilities come up that are expensive for the urban poor and yet the quality of their services is not guaranteed^12^.

Language barrier affected the utilization of services by the refugee girls. The health education sessions at health facilities are conducted in English and Luganda. None of these languages however is native to the refugees. This finding concurs with another study that stipulated language barrier as a challenge to offering health services to refugees10. The Government should rethink and redesign the health system and service delivery pack- age to cater for the urban refugees.

We concluded that although the refugee adolescents who get pregnant in Kampala are aware of the maternal health services offered, access and utilization of the services is still a challenge. Additionally, the standards for ANC were not met, while low attendance of PNC remains a challenge.

The refugee girls do not have the money to access and utilize maternal health services because most of them get pregnant while in school and those out of school have no jobs. Most of them do not have male support, and come from poor families.

In addition, Government health facilities are few and yet the private facilities that are accessible to the urban refugees have costly services. At the facilities some health workers are insensitive to the adolescent girls and this discourages them from interacting openly and returning. Additionally, there is overcrowding, language barrier, inadequate supplies and long waiting hours before they can receive the services and this discourages them from seeking the services.

The health system should be strengthened and more health workers recruited including those that can speak the languages that the refugees understand. Additionally, more funding should be allocated to adolescent-friendly service providers to ensure availability of all the drugs that are needed during pregnancy and delivery, as well as procurement of the necessary equipment like scans to enable the refugee adolescent girls to receive all the services required in one place.

These refugee girls have a right to safe and satisfying maternal health services. We therefore recommend that the government and implementing partners focus on how to improve service delivery and utilization for comprehensive SRHR services to the refugee adolescent girls.

We also recommend that further research is conducted to understand the vulnerabilities around the refugee adolescent girls who live in Kampala other urban areas in the country, the risk factors to sexual violence and how to protect them within their environments.
